# Managing shoulder pain in manual wheelchair users: a scoping review of conservative treatment interventions

**DOI:** 10.1177/0269215520917437

**Published:** 2020-05-12

**Authors:** Barry Mason, Martin Warner, Simon Briley, Victoria Goosey-Tolfrey, Riemer Vegter

**Affiliations:** 1 Peter Harrison Centre for Disability Sport, School for Sport, Exercise and Health Sciences, Loughborough University, Loughborough, UK; 2 School of Health Sciences, University of Southampton, Southampton, UK; 3 Arthritis Research UK Centre for Sport, Exercise and Osteoarthritis, University of Southampton, Southampton, UK; 4 Faculty of Medical Sciences, University of Groningen, Groningen, The Netherlands

**Keywords:** Shoulder pain, wheelchair, rehabilitation interventions

## Abstract

**Objective:**

To review the literature that has explored conservative treatments for the management of shoulder pain in manual wheelchair users.

**Methods:**

Five databases were systematically searched in february 2020 for terms related to shoulder pain and manual wheelchair use. Articles were screened and included if they investigated the conservative treatment of shoulder pain in wheelchair users. Participants’ physical characteristics, experimental design and primary and secondary outcome measures were extracted from studies. Studies were grouped according to treatment type to identify gaps in the literature and guide future research.

**Results:**

The initial search identified 407 articles, of which 21 studies met the inclusion criteria. Exercise-based treatment interventions were most prevalent (*n* = 12). A variety of exercise modalities were employed such as strengthening and stretching (*n* = 7), ergometer training (*n* = 3), Pilates classes (*n* = 1) and functional electrical stimulation (*n* = 1). Only three studies supplemented exercise with an additional treatment type. The Wheelchair Users Shoulder Pain Index was used by 18 studies as the primary measure of shoulder pain. Only seven of these included an objective measure of shoulder function. Participant characteristics varied among studies, and physical activity levels were frequently not reported.

**Conclusions:**

Despite the high prevalence of shoulder pain in manual wheelchair users, the number of studies to have explored conservative treatment types is low. Exercise is the most commonly used treatment, which is encouraging as physical inactivity can exacerbate other health conditions. Few studies have adopted interdisciplinary treatment strategies or included objective secondary measures to better understand the mechanisms of pain.

## Introduction

Manual wheelchair use places considerable stress on the upper limbs, particularly the shoulder, due to the repetitive loading induced by wheelchair propulsion in addition to other activities of daily living, such as transferring and weight relief tasks. Given the limited muscle mass and low stability, yet high mobility of the shoulder girdle,^1^ these activities often lead to pain, with up to 71% of manual wheelchair users reported to have experienced shoulder pain at some point in their life.^2–4^

The most common pathologies associated with shoulder pain are shoulder impingement syndrome, rotator cuff tears and tendinopathy, bursitis, joint oedema and glenohumeral instability.^5–7^ The consequences of such pathologies can be incredibly severe for wheelchair users, as it may prevent individuals from being physically active, which can negatively affect their independence and quality of life.^8,9^ This lack of physical activity can also lead to secondary health conditions such as obesity and cardiovascular disease.^10^ Structural changes as a result of injury within the shoulder may also develop into chronic conditions such as osteoarthritis, where joint degeneration can take place and may ultimately require shoulder arthroplasty to repair.^11^ Such invasive, surgical techniques are not without risk and should be considered a last resort given the prolonged postoperative immobilization imposed.^12^

A variety of conservative treatment options are available as an alternative to surgery for the management of shoulder pain, including exercise, massage, electrical nerve stimulation, neuromuscular retraining and corticosteroid injections.^13^ Conservative treatment has shown to have beneficial effects on shoulder pain in non-wheelchair users; however, evidence is rated as low quality.^14^ In addition, it cannot be assumed that treatments for non-wheelchair users will also be appropriate for wheelchair users due to differences in upper and lower limb function, perceptions of pain and tasks of everyday life that might be affected by shoulder pain. A systematic review on treatment options for wheelchair users found positive outcomes on shoulder pain following conservative treatment.^15^ However, this review only explored the effectiveness of exercise-based treatments and concluded that exercise was important for managing shoulder pain without being able to offer suggestions on type, frequency or duration of exercise. Considering the varied nature and range of conservative treatments available, it is important to consider all options in addition to exercise to help determine the most appropriate treatment. Subsequently, the aim of the current scoping review was to map the existing literature that has explored conservative, noninvasive solutions for the treatment of shoulder pain in manual wheelchair users to identify gaps in the evidence base and to direct future research in this area.

## Methods

The scoping review was conducted according to previously developed guidelines.^16,17^ The selection process of identification, screening, eligibility and inclusion was performed in accordance to the Preferred Reporting Items for Systematic Reviews and Meta-Analyses (PRISMA) guidelines for scoping reviews.^18^

### Data sources and systematic search

An initial search of relevant databases (MEDLINE, PubMed, PsychINFO, SPORTDiscus and Web of Science) was performed using ‘shoulder’ AND ‘pain’ AND ‘wheelchair’ as the search terms. Having reviewed the abstracts of the studies identified by this initial search, it was decided that the terms ‘pathology’ (patholog*) and ‘injury’ (injur*) were also added to the search (please refer to the Supplementary material available online). The search was conducted in February 2020 using the aforementioned databases to identify studies published up until the end of January 2020. The reference lists of suitable studies and review papers identified by the search were also examined to identify any additional records.

### Study selection

The following inclusion/exclusion criteria were applied to determine the eligibility of the identified articles, developed by B.M., R.V. and M.W.:

#### Inclusion criteria

- Manual wheelchair users with shoulder pain
- All ages, genders, health conditions and activity levels
- Research design must include a conservative treatment intervention – either longitudinal or within-subject measures

#### Exclusion criteria

- Case reports or review articles
- Not available in English
- Involve invasive/surgical procedures

Studies identified by the search strategy were imported into Mendeley reference management software where any duplicate articles were removed. The titles and abstracts of all studies were reviewed by one author (B.M.) and evaluated against the eligibility criteria. A second reviewer (S.B.) performed the same process on a random sample of 25% of the articles, with a concordance of 98% between included and excluded articles. Where an agreement was not reached, the article proceeded to full-text review where all articles were examined by two authors independently (B.M. and M.W.). The level of agreement between the two authors after the first review was 96%. Articles that resulted in a disagreement were then revisited and resolved by direct communication between authors.

### Data extraction and synthesis

A database was developed in Microsoft Excel to document and assimilate extracted data from all included studies. Database design was agreed by B.M., R.V. and M.W., and the list of extraction categories is detailed below:

1) Author(s);
2) Year of publication;
3) Purpose;
4) Population characteristics (age, disability, years of manual wheelchair use, physical activity and sample size);
5) Methodology and design;
6) Type of intervention;
7) Duration of the intervention;
8) Outcome measures.

Two authors (B.M. and M.W.) then extracted data from all articles. An independent reviewer (S.B.) then checked 20% of both authors extractions for accuracy. Studies were then grouped and reported according to the type of intervention performed.

## Results

**Figure 1.**
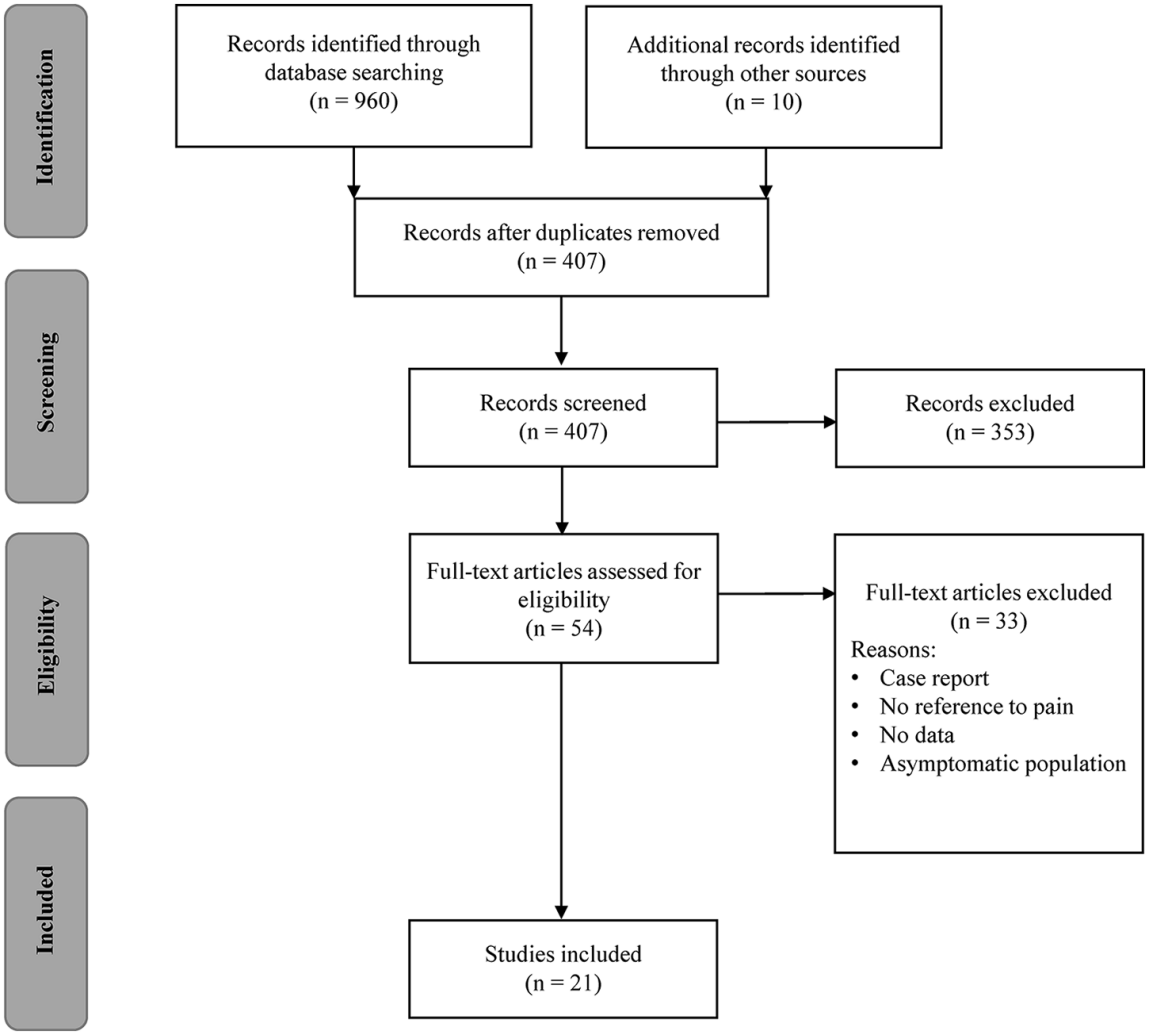
PRISMA flow diagram of the study selection process.

Of the 407 articles identified by the initial search, a total of 21 studies met the inclusion criteria (Figure 1). Studies were categorized according to the type of conservative treatment intervention. The most common treatment intervention was exercise-based (Table 1), which formed 12 of the 21 studies included.^14,19–29^ Home-based strengthening and stretching programmes were the most common modality of exercise prescribed (7/12 studies). Cardiovascular ergometer training was prescribed by three studies.^14,21,25^ Other studies explored strengthening and stretching in the form of supervised Pilates classes^26^ and functional electrical stimulation assisted rowing.^28^ The remaining studies were categorized as therapeutic-based (3/21),^30–32^ which included acupuncture, Trager Psychophysical Integration and transdermal nitroglycerine patches, equipment-based (1/21),^33^ and educational interventions (2/21),^34,35^ or interventions associated with lifestyle (3/21) assistance^36–38^ (Table 2). The majority of interventions were monodisciplinary. An interdisciplinary treatment approach was adopted by only three studies, where exercise was accompanied by either movement retraining or real-time electromyographical biofeedback.^22–24^

**Table 1.** Exercise-based interventions for the treatment of shoulder pain in manual wheelchair users.

Authors	Intervention	Duration (weeks)	Participants					Measures		Design
			Sample (*n*)	Age (years)	Disability	Experience (years)	Activity (hour/week)	Pain	Secondary	
Curtis et al.^19^	HEP strengthening and stretching. 3 × 15 reps daily	24	42 35 M; 7 F	35 ± 8	SCI, CP, MS & amputees	14 ± 9	Comm X- 12	PC-WUSPI	n/a	RCT
Dyson-Hudson et al.^14^	Arm-crank ergometer training. 3 × 20 min/wk	12	23 19 M; 4 F	41 ± 9	SCI (tetra & para)	15 ± 9	Comm 5 ± 4	WUSPI	n/a	RCT
Garcia-Gomez et al.^29^	HEP strengthening and stretching. 3 × 30 min/wk	10	36 15 M; 21F	26 ± 8	Not stated	Not stated	Athletes > 6	SPI-WB	Impingement tests & RoM	Quasi
Kemp et al.^22^	HEP & movement training 3/wk vs. 1-hr educational video	12	58 Not stated	22-72	SCI (all para)	20 ± 11	Comm Not stated	WUSPI	n/a	RCT
Middaugh et al.^24^	HEP & EMG biofeedback. 4/wk exercise. 5 EMG sessions	12	15 12 M; 3 F	23-56	SCI (tetra & para)	X- 16	Not stated	PC-WUSPI	n/a	RCT
Mulroy et al.^23^	HEP & movement training 3/wk vs. 1-hr educational video	12	58 Not stated	45 ± 11	SCI (all para)	22 ± 12	Comm Not stated	WUSPI	Shoulder torque & RoM	RCT
Nash et al.^21^	Resistance & arm-crank ergometer. 3 × 45 min/wk	16	7 7 M; 0 F	39-58	SCI (all para)	13 ± 7	Comm Not stated	WUSPI	Strength & power	Coh
Nawoczenski et al.^20^	HEP strengthening and stretching daily	8	41 28 M; 13 F	47 ± 12	SCI (tetra & para)	17 ± 13	Comm Not stated	PC-WUSPI	n/a	Quasi
Norrbrink et al.^25^	Double-poling ergometer training	10	8 6 M; 2 F	51 ± 11	SCI (all para)	18 ± 8	Comm Not stated	WUSPI	n/a	Coh
Van der Linden et al.^26^	Supervised Pilates classes. 1–2 × 60 min/wk	12	15 8 M; 7 F	51 ± 8	MS	Not stated	Comm Not stated	VAS	Interscapular distances	Coh
Van Straaten et al.^27^	HEP strengthening and stretching. 3 × 30 reps, 3/wk	16	16 13 M; 3 F	25-64	SCI/polio	X- 16	Comm Not stated	WUSPI	Isometric strength	Coh
Wilbanks et al.^28^	FES assisted-rowing programme. 3 × 30 min/wk	6	10 8 M; 2 F	47 ± 12	SCI (all para)	18 ± 14	Comm Not stated	WUSPI	Isokinetic strength, EMG	Coh

HEP: home exercise programme; EMG: electromyography; FES: functional electrical stimulation; SCI: spinal cord injury; tetra: tetraplegia; para: paraplegia; CP: cerebral palsy; MS: multiple sclerosis; Comm: community users; RCT: randomized controlled trial; Coh: cohort; Quasi: quasi-experimental; WUSPI: wheelchair users shoulder pain index; PC-WUSPI: performance-corrected wheelchair users shoulder pain index; VAS: visual analogue scale; RoM: range of movement.

**Table 2.** Additional treatment interventions conducted in manual wheelchair users with shoulder pain.

Authors	Intervention	Duration (weeks)	Participants					Measures		Design
			Sample (*n*)	Age (years)	Disability	Experience (years)	Activity (hour/week)	Pain	Secondary	
Therapeutic:										
Dyson-Hudson et al.^30^	Acupuncture vs. TPI. 10 treatments over 5 weeks	15	18 14 M; 4 F	45 ± 11	SCI (tetra & para)	15 ± 8	Comm 6 ± 7	PC-WUSPI	n/a	Quasi
Dyson-Hudson et al.^31^	Acupuncture vs. placebo. 10 treatments over 5 weeks	15	17 15 M; 2 F	39 ± 11	SCI (tetra & para)	11 ± 9	Comm 8 ± 13	PC-WUSPI	n/a	RCT
Giner-Pasqual et al.^32^	Transdermal nitroglycerine patch vs. placebo. Daily	24	41 Not stated	42-54	SCI (all para)	Not stated	Athletes Not stated	WUSPI	RoM	RCT
Equipment:										
Finley and Rodgers^33^	2-geared, nonpowered MAGIC Wheels – 5 months	28	13 7 M; 6 F	46 ± 14	SCI/polio	15 ± 10	Not stated	PC-WUSPI	Impingement tests & RoM	Coh
Educational:										
Hoenig et al.^34^	Education on fitting & propulsion vs. standard care	24	57 Not stated	65 ± 14	Not stated	13 ± 7	Comm Not stated	Yes / No	n/a	
Rice et al.^35^	Upper limb preservation guidance vs. standard care	52	37 28 M; 9 F	38 ± 16	SCI (tetra & para)	Not stated	Comm Not stated	PC-WUSPI	Propulsion kinetics	RCT
Lifestyle:										
Hubert et al.^36^	19 days training with mobility service dog	28	11 Not stated	Not stated	SCI (not stated)	Not stated	Comm Not stated	WUSPI	n/a	Coh
Vincent et al.^37^	Mobility service dog to provide lifestyle assistance	54	66 45 M; 21 F	X- 41	SCI (not stated)	Not stated	Comm Not stated	WUSPI	n/a	Coh
Vincent et al.^38^	Mobility service dog to provide lifestyle assistance	54	17 9 M; 8 F	42 ± 15	SCI (not stated)	Not stated	Comm Not stated	WUSPI	n/a	Coh

TPI: Trager Psychophysical Integration; SCI: spinal cord injury; tetra: tetraplegia; para: paraplegia; Comm: community users; RCT: randomized controlled trial; Coh: cohort; Quasi: quasi-experimental; WUSPI: wheelchair users shoulder pain index; PC-WUSPI: performance-corrected wheelchair users shoulder pain index; RoM: range of movement.

Sample sizes ranged from as little as seven participants^21^ to as many as 66 participants.^37^ The age range of participants was quite spread, yet similar across studies. Manual wheelchair users with a wide range of health conditions were included in the studies, including individuals with both paraplegia and tetraplegia as well as amputations and neuromuscular impairments. Years’ experience of manual wheelchair use was also quite spread, although similar across studies, yet not reported by all. The physical activity levels of participants was only provided by five studies, and the level of detailed was limited where only hours per week were typically reported.

Of the included studies, 11 adopted an experimental study design, of which eight were randomized control trials and three were quasi-experimental. The remaining 10 studies were observational prospective cohort studies. Interventions lasted from as little as six weeks up to as much as 12 months. All but three studies^26,29,34^ measured shoulder pain according to the Wheelchair Users Shoulder Pain Index, of which seven reported a performance corrected version of this questionnaire.^19,20,24,30,31,33,35^ Only nine studies included an objective measure of shoulder function, such as strength, range of movement and muscular activity.

## Discussion

The current systematic scoping review revealed that a total of 21 studies have investigated conservative treatment interventions for managing shoulder pain in wheelchair users. This is considerably lower than a similar review conducted in non-wheelchair users, where 177 studies were identified.^13^ This illustrates the paucity of research specific to manual wheelchair users and highlights the need for an increase in well-designed studies investigating the conservative treatment of shoulder pain, given the high prevalence within this population.^2–4^

### Treatments

Exercise-based interventions were the most popular type of treatment. The majority involved a programme of strengthening and stretching exercises using elastic training bands or weights.^19,20,21–24,27,29^ Arm-crank^14,21^ and double-poling^25^ ergometry interventions were also trialled, in addition to rowing assisted with functional electrical stimulation as additional means for strengthening rotator cuff muscles.^28^ One study used an alternative approach to reducing shoulder pain by focusing less on the shoulders and more on core strengthening through a Pilates exercise programme.^26^ The structure and supervision provided by exercise classes, such as Pilates, could prove to be a topic worthy of future investigation due to issues around adherence in home-based exercise programmes. Activity logs implemented by two studies noted that good adherence (>75% of all sessions completed) was only reported in 36% to 73% of participants during home-based exercise programmes.^20,27^ Programme duration (six weeks to six months) and frequency of exercise (daily to three times/week) also varied among studies. Subsequently, further work is required to determine not only the optimal type but also the dosage of exercise prescribed when attempting to reduce shoulder pain.

Aside from exercise, therapeutic interventions were the second most popular choice of treatment within the scientific literature, although only three such studies were performed.^30–32^ These studies explored the use of acupuncture,^30,31^ Trager Psychophysical Integration,^30^ and transdermal nitroglycerine patches.^32^ Acupuncture refers to the insertion of fine needles into specific locations around the body to correct energy flow imbalances thought to lead to pain and illness.^30^ Trager Psychophysical Integration is a technique that involves hands-on manipulation and movement re-education, anecdotally thought to minimize joint pain and improve mobility in individuals with a musculoskeletal disorder.^30^ Finally, transdermal nitroglycerine patches emit nitroglycerine through the skin, which is transformed into nitric oxide in the bloodstream and has been reported to be advantageous for the repair and regeneration of damaged tendons.^39,40^ However, detrimental side effects, such as headaches, were frequently reported with this type of treatment.^32,39,40^ Irrespective of the effectiveness of these individual treatment types, a broad range of therapeutic options exist, such as massage, manual therapy and corticosteroid injections,^13^ that have yet to be explored in manual wheelchair users and could be worthy of future investigation. It was noted that three studies had explored the effectiveness of gluco-corticoid or corticosteroid injections. However, these had to be excluded from the review since each study was a single sample case report, which did not satisfy the inclusion criteria.

The remaining six studies explored equipment,^33^ educational^34,35^ and lifestyle assistance^36–38^ interventions. The only study to investigate equipment-based interventions, studied the effect of two-geared MAGIC Wheels on shoulder pain.^33^ The gearing system of MAGIC Wheels allows participants to select between two different diameter push rims, depending on the task and can subsequently minimize the force and frequency of pushes performed by the user.^33^ Hoenig et al.^34^ and Rice et al.^35^ explored the effects of educating users on aspects such as wheelchair fitting, technique and upper limb preservation. However, it could be argued that this type of specialist education and training is best provided to prevent shoulder pain rather than as a treatment. Three studies examined the use of mobility service dogs for managing shoulder pain in wheelchair users.^36–38^ Mobility service dogs can be secured to the front or side of a wheelchair to pull the user and assist with activities of daily living that can be challenging when experiencing pain, such as pushing uphill, over rough terrain or negotiating kerbs.^38^ Concerns over the lack of cardiorespiratory stimulation reported when using a mobility service dog and the implications of such must be acknowledged.^41,42^ Therefore, this type of intervention could be of greater use to users suffering from severe shoulder pain to help maintain their independence, since the lack of physical activity experienced while using a mobility service dog could lead to other contraindications and health problems.

A lack of physical activity and cardiorespiratory stimulation could actually be a common issue associated with a number of the non-exercise-based interventions. Subsequently, interdisciplinary approaches may be advisable in the management of shoulder pain, which has previously been advocated for the preservation of upper limb function.^13,43^ However, very few studies identified by the current review adopted interdisciplinary interventions. Kemp et al.^22^ and Mulroy et al.^23^ both included ‘movement optimisation’ training alongside strengthening and stretching. The ‘movement optimisation’ training consisted of a series of recommendations provided by physical therapists to optimize skills that often provoke shoulder pain in wheelchair users (namely, wheelchair propulsion and transfers) and received frequent reinforcement on these tasks over the duration of the programme.^22,23^ Middaugh et al.^24^ utilized electromyographical biofeedback sessions to accompany the home exercise programme they had prescribed. Individuals who report musculoskeletal pain during repetitive tasks often struggle with the ‘rest’ part of the cycle where muscle relaxation is required.^44^ Subsequently, electromyographical biofeedback could be used to assist with muscle retraining and effectively relax overactive muscles during repetitive tasks such as wheelchair propulsion.^24^ Although biofeedback would appear a potentially feasible means for the treatment of shoulder pain, it remains to be seen whether this is a clinically viable option since access to specialist electromyographical equipment is unlikely to be widespread. That said, more studies of this nature attempting to incorporate other treatment modalities alongside an exercise-based programme are encouraged for the management of shoulder pain in wheelchair users.^13,43^

### Participants

Studies included participants with varied physical characteristics. The majority of studies were male dominant, and although a broad range of disabilities were investigated across studies, most focused on a specific health condition, rather than combining multiple. Although this approach guarantees homogeneity among participants to maximize internal validity, it can do so at the expense of external validity. This can cause problems for clinicians, as it prevents them and other practitioners from understanding which populations certain treatments may be generalized to.

The age range of participants was very broad, which implies that wheelchair users of varying experience levels have been accounted for; however, this information was not always provided. Future research must include details about the number of years participants have been using a manual wheelchair when examining shoulder pain, as different treatment types may be more appropriate for someone who has recently acquired an injury compared to someone who has spent numerous years pushing a wheelchair. This also raises another point for future consideration. Although it was not an original criterion for data extraction, studies should also consider how long participants have been experiencing pain, as again different treatment options may be required for acute and chronic symptoms. Many studies referred to this; however, as a bare minimum, future studies must include more detailed information regarding participants physical characteristics to assist clinicians with the treatment of shoulder pain for specific populations.

Another characteristic frequently not reported by studies was the physical activity levels of participants. Recreational activities outside of those performed for daily living could also predispose to a certain treatment type being more effective than another. For instance, sedentary individuals may respond better to an exercise-based treatment programme, whereas for individuals already accustomed to exercise, this might not be the case. Only one study identified by the current review investigated wheelchair athletes.^29^ During the initial search, a further two studies were identified that sampled wheelchair athletes.^45,46^ However, one study was excluded since it included wheelchair athletes asymptomatic of shoulder pain and used changes in shoulder range of motion to infer changes in pain rather than a direct measure,^46^ whereas the second study was a one sample case study with a paratriathlete.^45^ Although mixed findings have previously been reported as to whether wheelchair athletes are at a greater or reduced risk of developing shoulder pain than nonathletic wheelchair users,^47–49^ musculoskeletal differences are likely between these two populations as a result of their differing physical workloads. Subsequently, it should not be assumed that effective treatment methods for one population would be transferable to another and, in particular, athletic populations require further research.

### Measures

The Wheelchair Users Shoulder Pain Index was by far the most common tool used to quantify shoulder pain and was used by 18 of the 21 studies. Of the three studies not using this questionnaire, Hoenig et al.^34^ simply quantified shoulder pain as nominally present or not, whereas Van der Linden et al.^26^ and Garcia-Gomez et al.^29^ adopted an alternative visual analogue scale questionnaire. The use of a nominal scale fails to account for the magnitude of pain, which should be an important consideration for interventions. Given that the Wheelchair Users Shoulder Pain Index has been established as a valid and reliable instrument for reporting shoulder pain in wheelchair users,^50^ it is recommended that this questionnaire is reported to quantify pain wherever possible preferably in its performance corrected format. The performance corrected version is more applicable to all impairment types of wheelchair users since not all impairment types may perform all 15 activities themselves, and by performing a correction, comparisons can be made between individuals and studies if necessary.^4^ Clinicians would then be able to compare the relative effectiveness of different treatment options.

Although the Wheelchair Users Shoulder Pain Index is a good clinical tool for monitoring self-reported shoulder pain, pain itself can be considered a relatively subjective concept. Subsequently, future studies would be encouraged to include more objective measures of shoulder function alongside the presence of pain. Measures including range of movement, strength, muscular activity and propulsion kinetics were explored pre- and post-intervention by a limited number of studies. These objective measures could enable an insight into the mechanisms responsible for either causing or reducing shoulder pain and may further facilitate the identification of effective conservative treatment types for clinicians.

### Design

Of the available literature, nine of the 21 studies included randomized control trials. Although the aim of the current review was to simply map the available literature and the methodological designs adopted, future research into the effectiveness of the treatment interventions adopted will be warranted. In that case, reliable cause and effect relationships between the treatment and its effect on shoulder pain are paramount, for which randomized control trials remain the gold standard.^51^ Although there are many challenges associated with implementing randomized control trials, such as cost, time and loss of participants to follow-up,^51^ more of these studies are required to establish the effectiveness of conservative treatment types for reducing shoulder pain in wheelchair users in future.

A limitation associated with the current study was that the effectiveness of each treatment type was not provided. Although this information could be extremely valuable for clinicians, to assist with their treatment selection, the current review was a scoping review designed to identify gaps in the literature to help stimulate further research. Subsequently, it was not appropriate to conduct a detailed appraisal of included studies design and quality, nor the effectiveness of the interventions, as would have been expected for a systematic review. That said, this is still something of interest for future research. A subsequent limitation may lie within the search terms or inclusion/exclusion criteria adopted. Treatments such as injections could not be documented since the limited number of studies conducted in wheelchair users were all case reports. The only study to explore shoulder pain in athletic wheelchair users was also a case report. Subsequently, future research should consider including single sample case reports so that clinicians can gain a broader understanding of effective treatment types and how they may differ in different wheelchair user populations.

In conclusion, despite the prevalence of shoulder pain among manual wheelchair users, previous research into conservative treatments to help manage this problem has been scarce. Future research would be recommended to adopt interdisciplinary/multifaceted interventions, with exercise at the heart of the study. Studies of this nature are important so that shoulder pain can be treated without neglecting other factors such as physical activity, which are equally important yet are often overlooked during monodisciplinary studies. Future studies must also report the physical characteristics of the participants investigated. These steps will enable clinicians to optimize their treatment strategies and to establish which strategies can be transferable to specific patients.

**Clinical messages**

- Exercise was the conservative treatment most frequently used to manage shoulder pain in wheelchair users.
- Few studies have explored multidisciplinary treatment strategies for reducing shoulder pain in wheelchair users.
- The Wheelchair Users Shoulder Pain Index was the commonly used tool for quantifying shoulder pain.

## Supplemental Material

Supplementary_Material – Supplemental material for Managing shoulder pain in manual wheelchair users: a scoping review of conservative treatment interventions Click here for additional data file. Supplemental material, Supplementary_Material for Managing shoulder pain in manual wheelchair users: a scoping review of conservative treatment interventions by Barry Mason, Martin Warner, Simon Briley, Victoria Goosey-Tolfrey and Riemer Vegter in Clinical Rehabilitation
