# Supplementary material for: Managing shoulder pain in manual wheelchair users: a scoping review of conservative treatment interventions
Source: Clin Rehabil. 2020 May 12;34(6):741–53. doi: 10.1177/0269215520917437 (PMC7364791; doi:10.1177/0269215520917437)
Supplement: Supplementary_Material – Supplemental material for Managing shoulder pain in manual wheelchair users: a scoping review of conservative treatment interventions [file Supplementary_Material.pdf]

Supplementary Material

Search strategy – 'SPORTDiscus with Full Text' (via EBSCOhost)

- Final search performed July 2019 by BM
- Box checked "also search within the full text of the articles"
- Year limit selected - 1st January 1990
- TI Title field:
  [wheelchair AND shoulder AND pain]
  [wheelchair AND shoulder AND disorder]
  [wheelchair AND shoulder AND dysfunction]
  [wheelchair AND shoulder AND pathology]
  [wheelchair AND shoulder AND patholog*]
  [wheelchair AND shoulder AND injury]
  [wheelchair AND shoulder AND injur*]
- AB Abstract field:
  [wheelchair AND shoulder AND pain]
  [wheelchair AND shoulder AND disorder]
  [wheelchair AND shoulder AND dysfunction]
  [wheelchair AND shoulder AND pathology]
  [wheelchair AND shoulder AND patholog*]
  [wheelchair AND shoulder AND injury]
  [wheelchair AND shoulder AND injur*]
- KW Keywords:
  [wheelchair AND shoulder AND pain]
  [wheelchair AND shoulder AND disorder]
  [wheelchair AND shoulder AND dysfunction]
  [wheelchair AND shoulder AND pathology]
  [wheelchair AND shoulder AND patholog*]
  [wheelchair AND shoulder AND injury]
  [wheelchair AND shoulder AND injur*]
